# Sources of error in measurement of minimal residual disease in childhood acute lymphoblastic leukemia

**DOI:** 10.1371/journal.pone.0185556

**Published:** 2017-10-03

**Authors:** Sue Latham, Elizabeth Hughes, Bradley Budgen, Francoise Mechinaud, Catherine Crock, Henry Ekert, Peter Campbell, Alexander Morley

**Affiliations:** 1 Department of Haematology and Genetic Pathology, Flinders University and Medical Centre, Bedford Park, SA, Australia; 2 Childrens Cancer Centre, The Royal Childrens Hospital, Parkville Vic, Australia; 3 Clinical Haematology Department, The Royal Childrens Hospital, Parkville Vic, Australia; 4 Cancer Genome Project, Wellcome Trust Sanger Institute, Hinxton, United Kingdom; German Cancer Research Center (DKFZ), GERMANY

## Abstract

**Introduction:**

The level of minimal residual disease (MRD) in marrow predicts outcome and guides treatment in childhood acute lymphoblastic leukemia (ALL) but accurate prediction depends on accurate measurement.

**Methods:**

Forty-one children with ALL were studied at the end of induction. Two samples were obtained from each iliac spine and each sample was assayed twice. Assay, sample and side-to-side variation were quantified by analysis of variance and presumptively incorrect decisions related to high-risk disease were determined using the result from each MRD assay, the mean MRD in the patient as the measure of the true value, and each of 3 different MRD cut-off levels which have been used for making decisions on treatment.

**Results:**

Variation between assays, samples and sides each differed significantly from zero and the overall standard deviation for a single MRD estimation was 0.60 logs. Multifocal residual disease seemed to be at least partly responsible for the variation between samples. Decision errors occurred at a frequency of 13–14% when the mean patient MRD was between 10^−2^ and 10^−5^. Decision errors were observed only for an MRD result within 1 log of the cut-off value used for assessing high risk. Depending on the cut-off used, 31–40% of MRD results were within 1 log of the cut-off value and 21–16% of such results would have resulted in a decision error.

**Conclusion:**

When the result obtained for the level of MRD is within 1 log of the cut-off value used for making decisions, variation in the assay and/or sampling may result in a misleading assessment of the true level of marrow MRD. This may lead to an incorrect decision on treatment.

## Introduction

Since the early reports [1–4] a number of studies using PCR or flow cytometry have shown that the level of minimal residual disease (MRD) in bone marrow during the early phase of treatment is strongly predictive of outcome in children with acute lymphoblastic leukemia (ALL). As a result, measurement of the level of MRD in marrow has become part of standard management of childhood ALL in order to predict outcome and hence make decisions on treatment. Treatment intensity has been increased in patients in whom the MRD result has predicted a poor outcome and, in some studies, has been decreased in patients in whom an undetectable MRD result has predicted an excellent outcome [5]. However, prediction has been incorrect in a minority of patients although it is unclear how often failure of prediction has been due to biological variation of the leukemia in the individual patient and how often failure has been due to the measured MRD level not providing an accurate measure of overall marrow MRD.

The accuracy of the measurement of MRD depends on both the precision of the assay and the precision of sampling. Errors in sampling would be expected if the distribution of MRD in the marrow is focal rather than diffuse. Mathé *et al* [6] reported an extensive histological and cytological survey of 31 patients with ALL who had been judged to be in remission on the basis of a normal diagnostic marrow aspirate. They detected focal disease in 6 patients. Sykes *et al* [7] compared levels of MRD in paired aspirate and trephine samples from 22 patients and found increasing discordance as MRD levels fell below 5 x 10^−4^. This was attributed to sampling error, which was suggested to be due to multifocal residual disease. However, Van der Velden *et al* [8] studied a group of 26 patients in whom bilateral paired aspirations had been performed at various times during therapy and concluded that the frequency and magnitude of sampling error made it unnecessary to analyse more than one sample.

In this study, 2 approaches were taken to studying the magnitude and frequency of error in MRD measurement. Firstly, the variation in MRD measurement was determined by quantifying the variation between assays, between samples obtained from the same local area of marrow, and between samples obtained from 2 widely separated areas of marrow. Secondly, from the individual MRD results we estimated the frequency with which an individual assay would potentially lead to an incorrect decision on treatment.

## Materials and methods

### Patients and samples

Forty-one children with B-lineage ALL were studied. Their ages ranged from 2 years to 17 years with a median age of 6 years; 63% were male and 37% were female. The study was approved by the Royal Childrens Hospital and Flinders Medical Centre Ethics Committees and parental informed consent was obtained. Induction treatment was with vincristine, prednisolone, daunorubicin and asparaginase. All aspirations were performed under general anesthesia on day 35 at the end of induction. A routine diagnostic aspiration from the posterior superior iliac spine on one side was first performed. For the study, 2 separate aspirations of approximately 0.5 ml were then performed, angling the 2 needles in different directions, followed by 2 separate aspirations from the iliac spine on the other side, again angling the 2 needles in different directions.

### Measurement of MRD [9]

For each sample MRD was measured twice, on 2 separate days by different individuals. MRD was quantified using one IGH target. Three rounds of PCR were used with sequential forward primers being directed to the V region, the N_1_ region and the N_2_ region. A single reverse primer was directed to the J region. The number of cycles for each PCR round and the dilutions between each round were such that amplification remained exponential until the final PCR, which was a quantitative real-time PCR using a Taqman probe. MRD was measured in 10 μg of DNA. This gave a level of approximately 10^−6^ for detection of a single target molecule and assays which gave a negative result were expressed as a value which was “less than” that MRD value which corresponded to one intact IGH target in the reaction tube. Each assay tested the patient primers for non-specific amplification from 10 μg of peripheral blood DNA pooled from 5 individuals without leukemia. The standard deviation of the assay was approximately 0.25 logs but it increased above this value when fewer than 10 targets were present in the assay owing to the stochastic Poisson effect which occurs when the MRD level is close to the limit of detection of the assay. The MRD results were logarithmically transformed for all analysis except where stated.

### Statistical analysis

The criteria for inclusion of a patient for analysis of variance (ANOVA) were: the mean of the MRD assays was > 2 x 10^−6^, and; MRD was quantifiable at least once in every sample, and; MRD was quantifiable in at least 6 of the 8 assays. Four-way nested ANOVA was used to quantify the amount of variance due to each of the nested levels namely: Patient, Side, Sample, and Assay. As a small proportion of the data (3%) from the 29 eligible patients had missing MRD measurements, the maximum likelihood estimator was used in Stata’s mixed procedure(1) with robust standard errors estimators to control for possible heteroscedasticity of errors [10, 11]. The nested random-effect model was fitted with four factors Patient, Side|Patient, Sample|Side and Assay|Sample. This model corresponds to the three-level random intercept multilevel model [12] that was estimated with the mixed procedure in Stata (mixed MRD || Patient_ID: || Side: || Sample:, vce(robust) level(99)). Outliers were investigated with the Grubbs' test [13, 14] and Tukey’s test [15]. The results of ANOVA are shown in S2 ANOVA.

### Incorrect decisions

MRD results from all 41 patients were analysed. To estimate the frequency with which a single assay might lead to an incorrect decision in relation to intensifying treatment, the mean of the 8 assays in each patient was taken as the best estimate of the true MRD value in marrow; 3 separate analyses were performed taking the cut-off value for high-risk as being MRD greater than either 10^−3^, 5 x 10^−4^ (log_10_ = -3.3) or 10^−4^; and an incorrect decision was scored when the individual assay provided a different estimate of risk than that provided by the mean MRD value, i.e., when the individual assay was below the cut-off value and the mean value was above the cut-off value or the individual assay was above the cut-off value and the mean value was below the cut-off value.

A moving average of the percentage of assays which resulted in a decision error in relation to the assay result was performed by calculating the difference between the assay MRD result and each cut-off value, ranking the differences together with their corresponding decisions and determining a 45-point moving average of the percentage of assays which gave a decision error.

## Results

There were 41 patients, 164 samples and 319 assays. All results are shown in **S1 MRD Levels**. Eight assays were performed in all 41 patients with the following exceptions: a second assay was inadvertently omitted for all 4 samples from 1 patient and, owing to insufficient DNA, was not possible for 3 samples from 1 patient and 1 sample from each of 2 patients. The median MRD for the 41 patients was 2.5 x 10^−5^. MRD could be detected and quantified in all 8 assays in 26 patients, in 1–7 of the assays in 10 patients and could not be detected in any assay in 5 patients. There was no significant difference (p>0.4) between the mean MRD value for aspirates from the 2 sides. Non-specific amplification from control DNA was not observed.

Twenty-nine patients had sufficiently high MRD levels to fulfil the criteria for ANOVA. This is shown in **S1 ANOVA** and a summary is shown in Table 1. Assay, sample and side variance all differed significantly (p < 0.01) from zero. The total standard deviation (SD) of a single assay was 0.60 logs. This value comprises side, sample and assay variation and its magnitude suggests that errors in making decisions on treatment may occur when an MRD result is within approximately 1 log of the critical value used for making a treatment decision based on the result.

**Table 1:** Analysis of variance in all 29 patients. Results are expressed in log_10_ mode.

	variance		
	estimate	std. error	99% confidence interval
between patients	1.143	0.229	0.681–1.916
between sides	0.261	0.202	0.036–1.90
between samples	0.023	0.013	0.005–0.097
between assays	0.077	0.016	0.045–0.131

A subgroup analysis of assay, sample and side variation was performed grouping mean patient MRD values into those > 10^−3^, 10^−3^–10^−5^, and < 10^−5^. All SDs differed significantly (p<0.01) from 0 except that for sample variation in patients with MRD > 10^−3^. The results are shown in Table 2.

**Table 2:** Analysis by ANOVA of sources of error in relation to MRD level. Results are expressed in log_10_ mode and the SD rather than the variance in S2 ANOVA is shown. All values of SD differed significantly (p<0.01) from 0 except that asterisked.

	MRD level	< 10^−5^	10^−5^–10^−3^	> 10−^3^
	number of patients	6	17	6
between sides	number of sides	12	34	12
	SD	0.30	0.23	0.75
between samples	number of samples	24	68	24
	SD	0.20	0.15	0.10*
between assays	number of assays	44	134	48
	SD	0.30	0.26	0.31
TOTAL	SD	0.47	0.38	0.82

*(p > 0.05).

**Fig 1:**
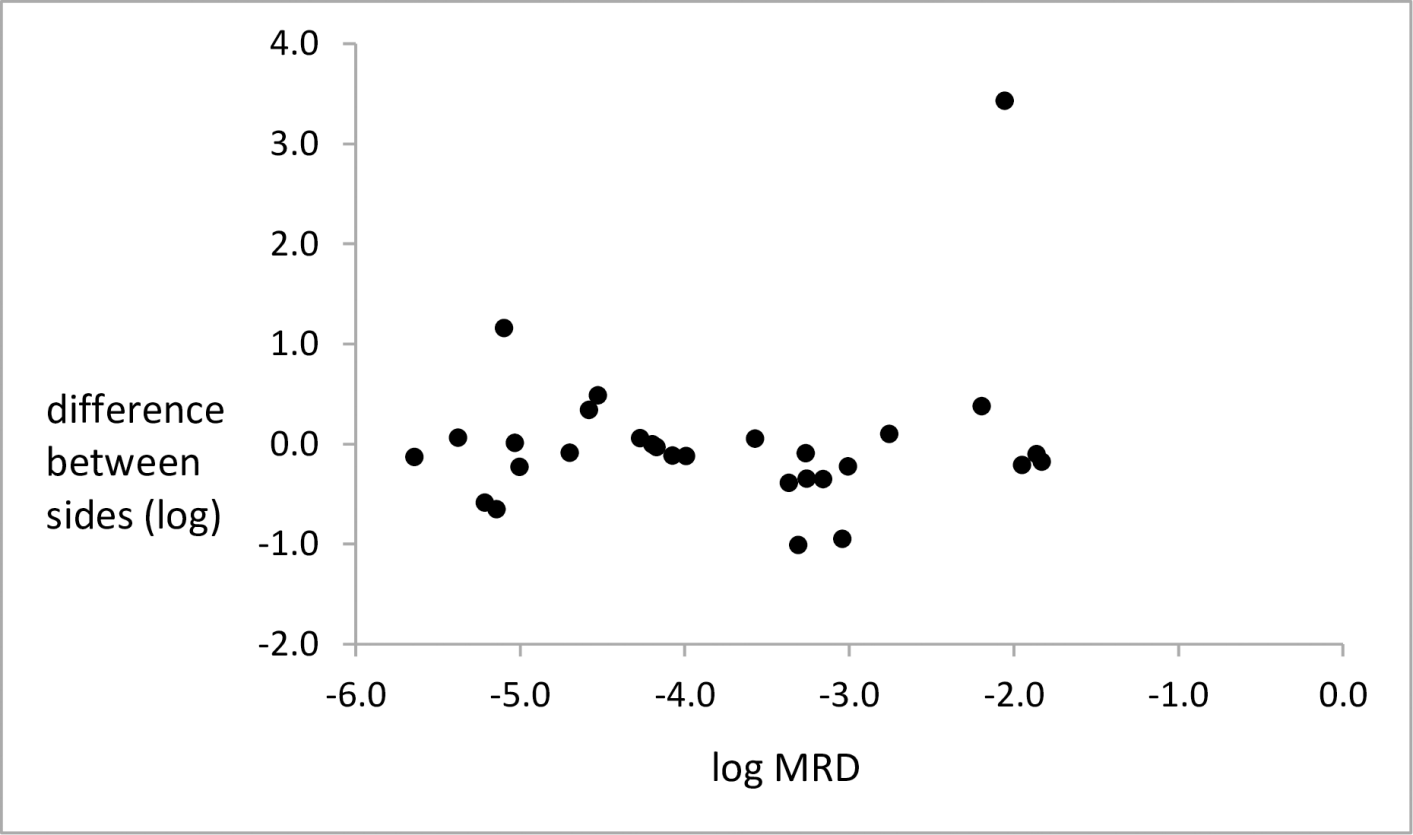
Difference between the left and right sides in the mean MRD value for each side. Each mean was the result of 2 assays on each of 2 samples. The results are for the 29 patients analysed by ANOVA.

Fig 1 shows the difference between the mean MRD value for the 2 sides.

The difference between the mean MRD value for the 2 sides was 0.95, 0.98 and 1.01 logs in 3 patients and 3.43 logs in another patient (patient 28). For patient 28 the difference between the means for the 2 sides was highly significant (p<0.001 on Grubb’s test, and outside the outer fence on Tukey’s test), indicating that this patient was an outlier from the other patients in terms of side variation. The results from patient 28 contributed very substantially to between-side variation for MRD > 10^−3^ and when they were excluded from the analysis the SD for all patients was 0.40, the SD for MRD > 10^−3^ was 0.30 and the between-side variation for MRD > 10^−3^ was no longer significant (p > 0.05).

The data from all 41 patients were used to study the accuracy of decision-making on the basis of an MRD estimation and cut-off values of either 10^−3^, 5 x 10^−4^ (log_10_ = -3.3) or 10^−4^. Table 3 shows the data on incorrect decisions.

**Table 3:** Incorrect decisions grouped by the mean MRD level of the patient. The percent of assays is the percent of decision errors in the assays from patients with MRD between 10^−2^ and 10^−5^. The results shown are data from all 41 patients.

mean MRD	incorrect decisions based on MRD criterion of			assays	patients
	10^−3^	10^−3.3^	10^−4^		
> -2	0	0	0	24	3
-2 to -3	4	4	2	24	3
-3 to -4	16	16	10	72	9
-4 to -5	0	0	10	60	8
-5 to -6	0	0	0	67	9
< -6	0	0	0	72	9
-2 to -5	20	20	22	156	20
% of assays	13%	13%	14%		

There were 20 patients with mean MRD between 10^−2^ and 10^−5^. Based on the cut-off value for high-risk being MRD greater than either 10^−3^, 5 x 10^−4^ or 10^−4^, assays, which would have led to an incorrect decision were observed in 13%, 13% and 14% respectively of the assays and occurred in 7, 8 and 8 patients respectively. There were 3 patients with mean MRD > 10^−2^ and 18 patients with mean MRD < 10^−5^ and none of their assays would have produced an incorrect decision.

**Fig 2:**
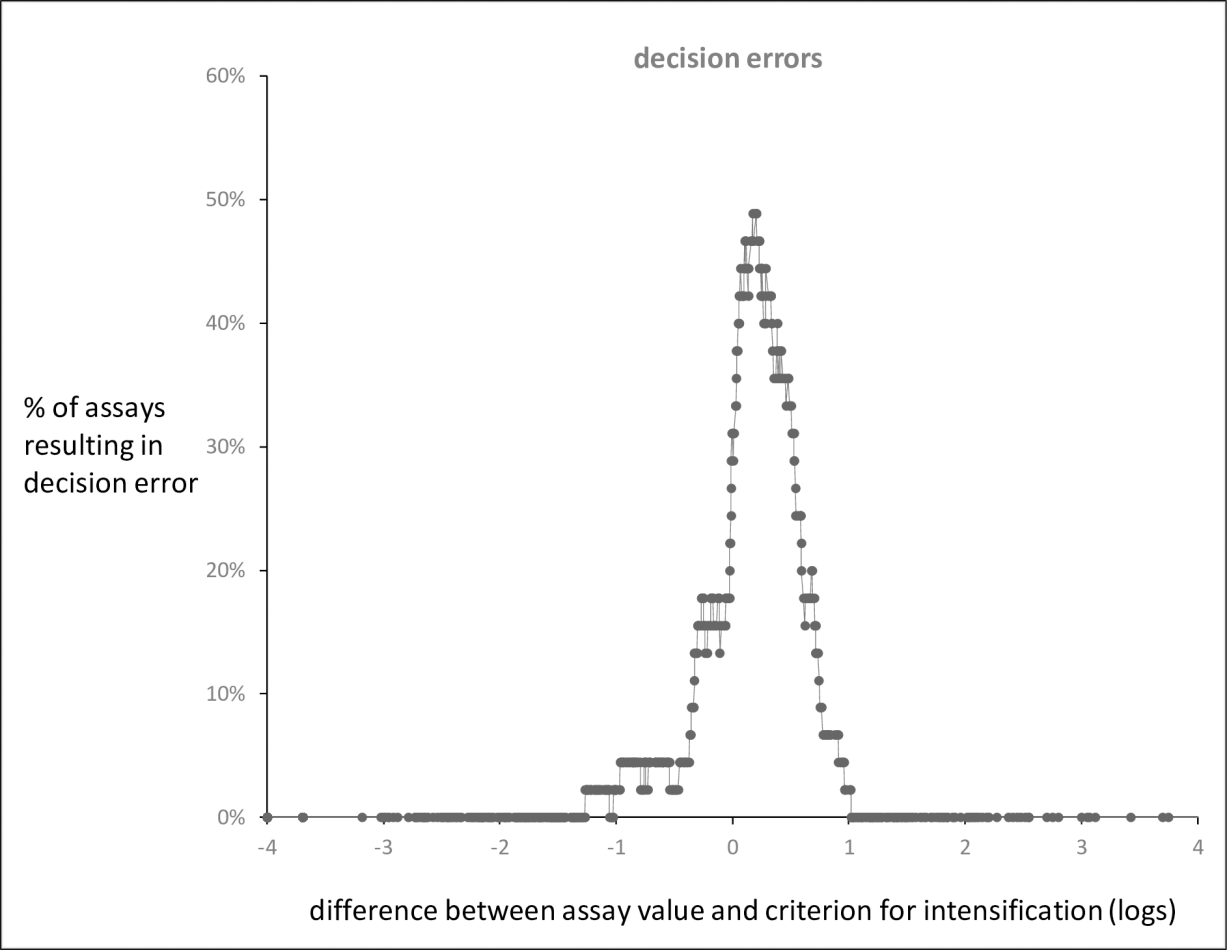
Percentage of decision errors in relation to difference between the assayed MRD value and the cut-off value used for deciding on intensification of treatment. There were 957 differences and the percentage of errors is a 45-point moving average. The results shown are data from all 41 patients.

For the 41 patients, there was a total of 957 decisions based on the assay result and the cut-off value for high risk being used. Fig 2 shows the percentage of incorrect decisions as a function of the difference between the assay result and the cut-off value.

The likelihood of a decision error increased markedly, and approached 50%, as the MRD result approached the cut-off value for decision. The percentage of assays within 1 log of each of the above criteria was 31%, 35% and 40% respectively and for these assays the percentage of decision errors was 21%, 18% and 16% respectively.

## Discussion

Using MRD levels to predict outcome and direct treatment is now part of standard management of childhood ALL, but prediction has still failed for many cases of that have relapsed. Biological variation is undoubtedly a factor in failure of prediction but inaccurate measurement of MRD is also likely to be important. Our data show that a single estimation of MRD is quite imprecise, and the overall data indicate that imprecision is significantly contributed to by variation between assays, by variation between samples taken from a local area, i.e., the one side, and by variation between samples taken from 2 distant areas, i.e., the 2 sides.

The magnitude of variation, as shown by the SD of 0.6 logs for a single assay, suggested that when the observed MRD value is within 1 log of the cut-off value used for decision-making, the errors in MRD estimation may be of sufficient frequency and magnitude to lead to errors in decisions on treatment. A decision error was scored when an individual MRD assay gave a different estimate of high risk than that given by the mean MRD value. In our 41 patients, the median MRD was 2.5 x 10^−5^ and MRD was between 10^−5^ and 10^−2^ in 49% of patients. Decision errors essentially occurred only in this group and in 13%-14% of assays in this range. However, in practice, when making a decision for an individual patient, the underlying MRD level is unknown and the decisions must be based on the result of the MRD assay. Fig 2 shows the likelihood of a decision error in relation to the deviation of the assay result from the MRD cut-off value for high risk. The results agree with above estimate of SD in suggesting that decision errors will tend to occur when the observed MRD value is within 1 log of the cut-off value. Assays within 1 log of the cut-off value used for decision comprised 31–40% of all assays and within this range the percentage of decision errors was 21%-16% and approached 50% as the assay result approached the cut-off value.

We therefore conclude that the magnitude of assay and sampling variation will lead to decision errors at a clinically important frequency when making treatment decisions based on MRD results. However, this conclusion needs to be considered in relation to the details of the treatment protocol being used. The MRD result is not the only factor in making decisions on treatment but it is the most important factor in most patients. The distribution of MRD values and the proportion of patients at risk of an incorrect decision will depend on the details of treatment. Assay of MRD may be performed twice on the same sample or on 2 samples obtained at different times. The precision of different PCR assay methods may differ and may depend on the MRD level. In some protocols MRD is measured by flow cytometry. This method will have its own variance but the material for assay will also be affected by sampling error.

In contrast to our conclusion, Van der Velden *et al* [8] concluded that the frequency and/or magnitude of sampling variation were such that it could in practice be ignored. They studied 26 patients and performed one aspiration from each side at various time-points; the potential limit of detection of their method was approximately 10^−5^; and the lower limit of the quantitative range of their method was either 10^−4^ or 5 x 10^−4^. For MRD below 10^−4^ they observed several possibly discordant results between the 2 sides, but the limited sensitivity of their method makes it impossible to draw any conclusion, as the results would have been affected by stochastic Poisson variation and possibly by non-specificity. For MRD above 10^−4^, the results for 41 paired samples obtained either on day 15, day 28, day 42 or at 3 months and in which quantitation was possible for both samples were presented in Fig 1B of their paper. This Figure shows that there were 5 or 6 pairs with MRD below 10^−3^ and 35 or 36 pairs with MRD above 10^−3^. None of the 5 or 6 pairs in the 10^−3^–10^−4^ range showed a material difference between the 2 samples but the small number of pairs and the fact that only 1 assay was performed on each sample make it impossible to draw any conclusion for MRD in this range. For the 35 or 36 pairs with MRD > 10^−3^ there were 2 pairs in which the results for the 2 members of the pair differed by more than a factor of 3. Differing by a factor of 3 is an indirect measure of variation.

Our data for MRD > 10^−3^ suggested significant between-assay and between-side variation in this group, but interpretation needs to take account of the small number of patients in this group and the presence of the outlying patient 28. We therefore feel that the importance of sampling error for patients with MRD > 10^−3^ remains an open question but we maintain our conclusion for patients with MRD < 10^−3^.

Variable dilution of marrow with blood may have contributed to between-sample variation, although precaution was taken to minimise this. However, it would not have contributed to the measure of between-side variation. The persistence of between-sample and between-side variation at all levels of MRD and particularly the correlation between the 2 samples on the same side suggests that distribution of MRD was often multifocal rather than being uniformly diffuse, a conclusion in agreement with the finding of Mathé *et al* [6]. Since side-to-side variation was seen at all levels of MRD, the samples obtained by the 2 differently-angled aspiration needles must sometimes have contained cells from the same leukemic focus, which in turn suggests that some foci can be sufficiently large to extend from close to the tip of one aspiration needle to the tip of the other angled aspiration needle. The results from patient 28 probably represent the extreme situation in which both aspiration needles on one side sampled the centre of a very large focus of leukemic cells. Leukemic cells at the end of induction are a pre-existing relatively-resistant subpopulation which has been selected out by chemotherapy [16], and it therefore seems likely that large foci are more likely to be present when the overall MRD level is high, rather than occurring randomly at any level of MRD.

In the light of our findings, what can or should be done to decrease potential error in MRD measurement at the end of induction? This question has been considered previously in relation to making decisions on treatment [17]. There is a series of options in which the negative factors of inconvenience, cost and patient morbidity are balanced against increased accuracy and precision and, potentially, improved patient outcome. One option is to accept the present level of error in MRD measurement and, in the light of the negative factors, to not change current practice. A variety of options which would decrease measurement variation would include, singly or in combination: performing multiple assays on the one sample, assaying more than one sample from the same side, assaying samples from the 2 sides, and pooling samples before assay. A simple option would be to perform 2 aspirations from the same side but in different directions and to assay each separately. A more speculative and long-term option would be to investigate the potential for quantification of MRD in a sample of blood [18–20] which, conceptually, samples all of the marrow in an unbiased fashion and which can be assayed at sufficient sensitivity by nested PCR or perhaps by next generation sequencing.

Finally, our results may have wider implications. In childhood ALL, if decreased intensity of treatment when the MRD level is very low were to become standard therapy, incorrect decisions might occur in an additional group of patients. In chronic lymphocytic leukemia and myeloma MRD is often measured in marrow and used to assess prognosis or guide treatment. However, in these 2 diseases, it is well recognised that the neoplastic cells in the marrow are often distributed multifocally and inaccurate measurement of MRD due to sampling error may therefore occur.

## Supporting information

S1 MRD Levels (DOCX)

S1 ANOVA (PDF)
