# Supplementary material for: Sources of error in measurement of minimal residual disease in childhood acute lymphoblastic leukemia
Source: PLoS One. 2017 Oct 3;12(10):e0185556. doi: 10.1371/journal.pone.0185556 (PMC5626434; doi:10.1371/journal.pone.0185556)
Supplement: S1 MRD Levels — (DOCX) [file pone.0185556.s001.docx]

PATIENTS MEETING CRITERIA FOR ANOVA

|  |  |  |  |  |  |  |  |
| --- | --- | --- | --- | --- | --- | --- | --- |
| Note: only 1 assay done for 6 samples | | |  |  |  |  |  |
| : logarithmic transformation of MRD values | | |  |  |  |  |  |
|  |  |  |  |  |  |  |  |
| patient number | left sample1 |  |  |  |  |  |  |
|  | left sample2 |  |  |  |  |  |  |
|  | right sample1 right sample2 |  |  |  |  |  |  |
|  |  |  |  |  | patient mean |  |  |
|  |  |  | means | | | difference |  |
| patient | assay1 | assay2 | assays | samples | sides | between sides |  |
|  |  |  |  |  |  |  |  |
| 1 | -5.18 |  | -5.18 | -5.34 | -5.38 | 0.06 |  |
|  | -5.51 |  | -5.51 |  |  |  |  |
|  | -5.16 |  | -5.16 | -5.41 |  |  |  |
|  | -5.65 |  | -5.65 |  |  |  |  |
| 2 | -2.80 | -2.91 | -2.85 | -2.71 | -2.76 | 0.10 |  |
|  | -2.54 | -2.58 | -2.56 |  |  |  |  |
|  | -2.77 | -2.95 | -2.86 | -2.80 |  |  |  |
|  | -2.82 | -2.68 | -2.75 |  |  |  |  |
| 3 | -5.45 | -5.95 | -5.70 | -5.71 | -5.64 | -0.13 |  |
|  | -5.72 | -5.70 | -5.71 |  |  |  |  |
|  | -5.26 | -5.58 | -5.42 | -5.57 |  |  |  |
|  | -5.54 | -5.92 | -5.73 |  |  |  |  |
| 4 | -4.21 | -4.01 | -4.11 | -3.82 | -3.31 | -1.01 |  |
|  | -3.54 | -3.49 | -3.52 |  |  |  |  |
|  | -3.05 | -2.72 | -2.88 | -2.80 |  |  |  |
|  | -2.82 | -2.62 | -2.72 |  |  |  |  |
| 5 | -3.75 | -3.57 | -3.66 | -3.56 | -3.36 | -0.39 |  |
|  | -3.42 | -3.51 | -3.46 |  |  |  |  |
|  | -2.90 | -3.02 | -2.96 | -3.17 |  |  |  |
|  | -3.34 | -3.41 | -3.38 |  |  |  |  |
| 6 | -2.97 | -3.29 | -3.13 | -3.12 | -3.01 | -0.23 |  |
|  | -3.04 | -3.19 | -3.11 |  |  |  |  |
|  | -2.97 | -3.22 | -3.10 | -2.89 |  |  |  |
|  | -2.61 | -2.77 | -2.69 |  |  |  |  |
| 7 | -3.60 | -3.66 | -3.63 | -3.33 | -3.16 | -0.35 |  |
|  | -3.19 | -2.89 | -3.04 |  |  |  |  |
|  | -3.16 | -2.75 | -2.96 | -2.98 |  |  |  |
|  | -3.25 | -2.76 | -3.00 |  |  |  |  |
| 8 | -3.63 | -3.29 | -3.46 | -3.31 | -3.26 | -0.10 |  |
|  | -3.26 | -3.07 | -3.16 |  |  |  |  |
|  | -3.06 | -3.18 | -3.12 | -3.21 |  |  |  |
|  | -3.36 | -3.26 | -3.31 |  |  |  |  |
| 9 | -4.19 | -3.90 | -4.05 | -4.05 | -3.99 | -0.12 |  |
|  | -4.09 | -4.01 | -4.05 |  |  |  |  |
|  | -3.75 | -4.01 | -3.88 | -3.93 |  |  |  |
|  | -4.01 | -3.94 | -3.98 |  |  |  |  |
| 10 | -1.82 | -2.28 | -2.05 | -1.91 | -1.86 | -0.10 |  |
|  | -1.46 | -2.10 | -1.78 |  |  |  |  |
|  | -1.48 | -2.16 | -1.82 | -1.81 |  |  |  |
|  | -1.55 | -2.04 | -1.80 |  |  |  |  |
| 11 | -5.96 | -5.68 | -5.82 | -5.47 | -5.14 | -0.66 |  |
|  | -5.45 | -4.79 | -5.12 |  |  |  |  |
|  | -4.82 | -4.77 | -4.80 | -4.82 |  |  |  |
|  | -5.01 | -4.66 | -4.83 |  |  |  |  |
| 12 | -4.25 | -3.95 | -4.10 | -4.24 | -4.27 | 0.05 |  |
|  | -4.37 | -4.39 | -4.38 |  |  |  |  |
|  | -4.34 | -4.52 | -4.43 | -4.29 |  |  |  |
|  | -4.26 | -4.06 | -4.16 |  |  |  |  |
| 13 | -4.52 | -4.86 | -4.69 | -5.03 | -5.03 | 0.01 |  |
|  | -5.64 | -5.09 | -5.36 |  |  |  |  |
|  | -5.16 | -4.84 | -5.00 | -5.03 |  |  |  |
|  | -5.01 | -5.12 | -5.06 |  |  |  |  |
| 14 | -4.06 | -3.79 | -3.93 | -3.52 | -3.04 | -0.95 |  |
|  | -3.17 | -3.04 | -3.11 |  |  |  |  |
|  | -2.58 | -2.58 | -2.58 | -2.56 |  |  |  |
|  | -2.72 | -2.37 | -2.55 |  |  |  |  |
| 15 | -3.61 | -3.91 | -3.76 | -3.54 | -3.57 | 0.05 |  |
|  | -3.24 | -3.41 | -3.32 |  |  |  |  |
|  | -3.60 | -3.84 | -3.72 | -3.60 |  |  |  |
|  | -3.47 | -3.48 | -3.48 |  |  |  |  |
| 16 | -5.06 | <-5.49 | -5.06 | -5.11 | -5.02 | -0.20 |  |
|  | -5.17 | <-5.72 | -5.17 |  |  |  |  |
|  | -4.56 | -4.55 | -4.56 | -4.92 |  |  |  |
|  | -5.46 | -5.10 | -5.28 |  |  |  |  |
| 17 | -1.98 | -2.35 | -2.16 | -1.92 | -1.83 | -0.18 |  |
|  | -1.45 | -1.89 | -1.67 |  |  |  |  |
|  | -1.85 | -1.20 | -1.53 | -1.74 |  |  |  |
|  | -1.98 | -1.92 | -1.95 |  |  |  |  |
| 18 | -2.39 | -2.22 | -2.31 | -2.05 | -1.95 | -0.21 |  |
|  | -1.80 | -1.81 | -1.80 |  |  |  |  |
|  | -1.95 | -1.59 | -1.77 | -1.84 |  |  |  |
|  | -2.09 | -1.73 | -1.91 |  |  |  |  |
| 19 | -5.22 |  | -5.22 | -5.12 | -5.00 | -0.23 |  |
|  | -4.30 | -5.73 | -5.01 |  |  |  |  |
|  | -4.34 | -5.24 | -4.79 | -4.89 |  |  |  |
|  | -4.97 | -5.00 | -4.99 |  |  |  |  |
| 20 | -3.72 | -4.55 | -4.13 | -4.20 | -4.19 | -0.01 |  |
|  | -4.14 | -4.39 | -4.26 |  |  |  |  |
|  | -3.86 | -4.39 | -4.13 | -4.19 |  |  |  |
|  | -4.03 | -4.48 | -4.26 |  |  |  |  |
| 21 | -4.77 | -4.75 | -4.76 | -4.74 | -4.70 | -0.09 |  |
|  | -4.60 | -4.85 | -4.72 |  |  |  |  |
|  | -5.14 | -4.36 | -4.75 | -4.65 |  |  |  |
|  | -4.61 | -4.50 | -4.55 |  |  |  |  |
| 22 | -3.84 | -4.52 | -4.18 | -4.13 | -4.07 | -0.12 |  |
|  | -3.93 | -4.24 | -4.09 |  |  |  |  |
|  | -3.80 | -4.44 | -4.12 | -4.01 |  |  |  |
|  | -3.75 | -4.06 | -3.91 |  |  |  |  |
| 23 | -4.57 | -4.27 | -4.42 | -4.52 | -5.01 | 0.98 |  |
|  | -4.67 | -4.56 | -4.61 |  |  |  |  |
|  | <-5.88 | -5.49 | -5.49 | -5.50 |  |  |  |
|  | -5.37 | -5.64 | -5.51 |  |  |  |  |
| 24 | -3.97 |  | -3.97 | -4.41 | -4.58 | 0.34 |  |
|  | -4.85 | -4.86 | -4.86 |  |  |  |  |
|  | -4.75 | -4.66 | -4.71 | -4.75 |  |  |  |
|  | -4.82 | -4.75 | -4.79 |  |  |  |  |
| 25 | -3.67 | -3.30 | -3.48 | -3.43 | -3.26 | -0.35 |  |
|  | -3.42 | -3.34 | -3.38 |  |  |  |  |
|  | -3.12 | -3.25 | -3.18 | -3.08 |  |  |  |
|  | -2.99 | -2.97 | -2.98 |  |  |  |  |
| 26 | -4.34 | -4.16 | -4.25 | -4.29 | -4.53 | 0.48 |  |
|  | -4.12 | -4.52 | -4.32 |  |  |  |  |
|  | -4.60 | -4.31 | -4.45 | -4.77 |  |  |  |
|  | -5.09 | -5.07 | -5.08 |  |  |  |  |
| 27 | -1.52 | -1.97 | -1.75 | -2.00 | -2.19 | 0.38 |  |
|  | -2.19 | -2.34 | -2.26 |  |  |  |  |
|  | -2.51 | -2.29 | -2.40 | -2.38 |  |  |  |
|  | -2.61 | -2.11 | -2.36 |  |  |  |  |
| 28 | -0.93 | -0.25 | -0.59 | -0.34 | -2.05 | 3.43 |  |
|  | 0.12 | -0.30 | -0.09 |  |  |  |  |
|  | -4.02 | -3.33 | -3.68 | -3.77 |  |  |  |
|  | -4.27 | -3.46 | -3.86 |  |  |  |  |
| 29 | -4.18 | -4.63 | -4.41 | -4.19 | -4.17 | -0.04 |  |
|  | -3.89 | -4.05 | -3.97 |  |  |  |  |
|  | -4.18 | -3.94 | -4.06 | -4.15 |  |  |  |
|  | -4.32 | -4.16 | -4.24 |  |  |  |  |
|  |  |  |  |  |  |  |  |
|  |  |  |  |  |  |  |  |

PATIENTS NOT MEETING CRITERIA FOR ANOVA

The “<” indicates that MRD was not detected and was below the MRD value corresponding to 1 target molecule in the assay. Such values cannot be logarithmically transformed.

| Patient # | MRD | |
| --- | --- | --- |
|  | 1st | repeat |
| 30 | <1.21E-06 | no DNA |
|  | <2.76E-06 | <1.39E-06 |
|  | <1.34E-06 | <9.36E-07 |
|  | <9.42E-07 | <9.92E-07 |
| 31 | <6.73E-07 | 1.15E-05 |
|  | <1.11E-06 | 8.59E-06 |
|  | 1.36E-06 | <8.53E-07 |
|  | 3.76E-06 | 6.22E-06 |
| 32 | <1.28E-06 | <1.17E-06 |
|  | 1.07E-06 | <1.03E-06 |
|  | <9.61E-07 | <1.25E-06 |
|  | <9.35E-07 | <1.26E-06 |
| 33 | <6.39E-07 | <1.32E-06 |
|  | <1.05E-06 | <1.34E-06 |
|  | <7.84E-07 | <1.58E-06 |
|  | <9.23E-07 | <9.31E-07 |
| 34 | <1.07E-06 | 2.72E-06 |
|  | <9.66E-07 | <6.38E-07 |
|  | <9.53E-07 | <8.85E-07 |
|  | <5.63E-08 | <9.76E-07 |
| 35 | <5.7E-6 | 3.70E-05 |
|  | <5.2E-6 | 4.60E-05 |
|  | 4.50E-06 | <1.7E-5 |
|  | <3.21E-6 | 5.04E-05 |
| 36 | <4xE-7 | <4xE-7 |
|  | <4xE-7 | <4xE-7 |
|  | <4xE-7 | <4xE-7 |
|  | <4xE-7 | <4x10-7 |
| 37 | 1.76E-05 | <2.19x10-7 |
|  | <2.4x10-6 | <2.84x10-7 |
|  | <2.5x10-6 | <2.2x10-7 |
|  | <1.5x10-6 | <2.2x10-7 |
| 38 | <1.63E-06 | 9.76E-06 |
|  | <8.44E-06 | 1.02E-05 |
|  | <3.32E-10-6 | 1.59E-05 |
|  | 2.00E-06 | 2.05E-05 |
| 39 | <2.0E-06 | <1.2E-06 |
|  | <1.4E-06 | <5.0E-07 |
|  | <5.7E-07 | <1.05E-06 |
|  | <5.7E-07 | <1.77E-06 |
| 40 | <1.23x10-6 | <1.23x10-6 |
|  | 1.50E-06 | <1.02x10-6 |
|  | <1.27x10-6 | <1.12E-06 |
|  | <1.27x10-6 | <9.83x10-7 |
| 41 | <3.28E-07 | <1.25E-06 |
|  | <8.66E-07 | <1.41E-06 |
|  | <1.65E-06 | <1.65E-06 |
|  | <1.01E-06 | <1.50E-06 |
