# Supplementary material for: Sources of error in measurement of minimal residual disease in childhood acute lymphoblastic leukemia
Source: PLoS One. 2017 Oct 3;12(10):e0185556. doi: 10.1371/journal.pone.0185556 (PMC5626434; doi:10.1371/journal.pone.0185556)
Supplement: S1 ANOVA — (PDF) [file pone.0185556.s002.pdf]

```
                                                                  ___  ____  ____  ____  ____(R)
                                                                 /__    /   ____/   /   ____/
                                                                ___/   /   /___/   /   /___/
                                                                  Statistics/Data Analysis

                                                                    User: Lincom
```

```
  ___  ____  ____  ____  ____ (R)
 /__    /   ____/   /   ____/
___/   /   /___/   /   /___/   14.1   Copyright 1985-2015 StataCorp LP
  Statistics/Data Analysis            StataCorp
                                      4905 Lakeway Drive
     Special Edition                  College Station, Texas 77845 USA
                                      800-STATA-PC        http://www.stata.com
                                      979-696-4600        stata@stata.com
                                      979-696-4601 (fax)

Single-user Stata perpetual license:
       Serial number:  401406224771
         Licensed to:  Statistical Consultant
                       Flinders University

Notes:
      1.  Unicode is supported; see help unicode_advice.
      2.  Maximum number of variables is set to 5000; see help set_maxvar.

1 . doedit "C:\! PRIVATE TO KEEP\! UNI TEMP\1290 # M_123_2016_04_08 # 40% EtbS\ADJUSTED PAPER\TABLE_1_TA

2 . do "C:\Users\zkuz0001\AppData\Local\Temp\STD00000000.tmp"

3 . do "C:\! PRIVATE TO KEEP\! UNI TEMP\1290 # M_123_2016_04_08 # 40% EtbS\ADJUSTED PAPER\ICCVAR\iccvar.

4 . *CALCUATES ICCs and Variances of ICCs based on Hedges Derivations
5 .
6 . program iccvar, rclass
  1.         syntax, [UNBalance] [Alpha(real 0.01)]
  2.         version 11.1
  3.         tempname cs l4vc l4vcv l3vc l3vcv l2vc l2vcv l1vc tv tv4 icc2 icc3 icc4 ns m N  ///
>         se2 se3 se4 v1 v2 v3 v4 b V p r q a b c d e f l u higher lower z cv23 cv24 ///
>         cv34 c23 icc2bar icc3bar icc4bar qp qp2 qp2tv4 qtv4
  4.         tempvar n
  5.         if e(cmd) == "xtmixed" | e(cmd) == "mixed" {
  6.                 local levels = 0
  7.                 local test1 = e(revars)
  8.                 foreach v in `test1' {
  9.                         if "`v'" == "_cons" {
 10.                                 local ++levels
 11.                         }
 12.                         else if "`v'" == "." {
 13.                                 display as error "must have at least one group-level random effec
 14.                                 exit 9
 15.                         }
 16.                         else {
 17.                                 display as error "only _cons can have random effects"
 18.                                 exit 9
 19.                         }
 20.                 }
 21.                 capture assert `levels' <= 3 & `levels' > 0
 22.                 if _rc == 9 {
 23.                         display as error "this command is for models with up to 4 levels only"
 24.                         exit 9
 25.                 }
 26.                 scalar `cs' = colsof(e(N_g))
 27.                 assert `cs' == `levels'
 28.
7 .                 *Display Headers
8 .
```

```
                display _newline as text "Intraclass Correlation Estimates" _newline

                if `levels' == 1 {
                        if "`unbalance'" == "unbalance" {
                                display as error "there is no balance option for two level models
                                exit 9
                        }
                        local l2var = e(ivars)
                        local y = e(depvar)
                        quietly : _diparm lns1_1_1, function((exp(@))^2) derivative(2*exp(@)^2)
                        scalar `l2vc' = r(est)
                        scalar `l2vcv' = (r(se))^2
                        quietly : _diparm lnsig_e, function((exp(@))^2) derivative(2*exp(@)^2)
                        scalar `l1vc' = r(est)
                        scalar `tv' = `l1vc' + `l2vc'
                        scalar `tv4' = ((`tv')^.5)^4
                        scalar `icc2' = `l2vc' / `tv'
                        scalar `v2' = (((1-`icc2')^2)*`l2vcv')/`tv4'
                        scalar `se2' = (`v2')^.5
                        matrix define `b' = (`icc2')
                        matrix define `V' = (`v2')
                        matrix colnames `b' = `l2var'
                        matrix rownames `b' = `y'
                        matrix colnames `V' = `l2var'
                        matrix rownames `V' = `l2var'
                }
                else if `levels' == 2 {
                        preserve
                        gen `n' = e(sample)
                        local lvars = e(ivars)
                        local k = 3
                        foreach v in `lvars' {
                                local l`k'var "`v'"
                                local --k
                        }
                        local y = e(depvar)
                        quietly : _diparm lns1_1_1, function((exp(@))^2) derivative(2*exp(@)^2)
                        scalar `l3vc' = r(est)
                        scalar `l3vcv' = (r(se))^2
                        quietly : _diparm lns2_1_1, function((exp(@))^2) derivative(2*exp(@)^2)
                        scalar `l2vc' = r(est)
                        scalar `l2vcv' = (r(se))^2
                        quietly : _diparm lnsig_e, function((exp(@))^2) derivative(2*exp(@)^2)
                        scalar `l1vc' = r(est)
                        scalar `tv' = `l1vc' + `l2vc' + `l3vc'
                        scalar `tv4' = ((`tv')^.5)^4
                        scalar `icc2' = `l2vc' / `tv'
                        scalar `icc3' = `l3vc' / `tv'
                        if "`unbalance'" == "" {
                                collapse (max) `n', by(`l2var' `l3var')
                                collapse (sum) `n', by(`l3var')
                                quietly : drop if `n' == 0
                                quietly: means `n'
                                scalar `p' = r(mean_h)

                                display as text "Harmonic Mean of Level 2 Units per Level 3 Unit" _c
                                return scalar p = `p'

                                *variance of icc2

                                scalar `v2' = (((`p'*((1-`icc2')^2))+(2*`icc2'*(1-`icc2')))*`l2vcv')
>
                                scalar `se2' = `v2'^.5

```

```
 15 .                                  *variance of icc3
 16 .
 17 .                                  scalar `v3' = ((((`p'*(`icc3'^2))+(2*`icc3'*(1-`icc3')))*`l2vcv') /
     86.                                  scalar `se3' = `v3'^.5
     87.
 18 .                                  *covariance
 19 .                                  scalar `cv23' = (((((`p'*`icc3'*(1-`icc2'))+(`icc2'*`icc3')+((1-`icc
    > (1-`icc3')*`l3vcv')/`tv4')
     88.
 20 .                                  matrix define `b' = (`icc3', `icc2')
     89.                                  matrix define `V' = (`v3', `cv23' \ `cv23', `v2')
     90.                                  matrix colnames `b' = `l3var' `l2var'
     91.                                  matrix rownames `b' = `y'
     92.                                  matrix colnames `V' = `l3var' `l2var'
     93.                                  matrix rownames `V' = `l3var' `l2var'
     94.
 21 .                          }
     95.                          else if "`unbalance'" == "unbalance" {
     96.
 22 .                                  *getting covariance between variance components
 23 .
 24 .                                  collapse (sum) `n' if e(sample), by(`l2var' `l3var')
     97.                                  quietly : gen a = `n'/((`n'*(`l2vc'))+(`l1vc'))
     98.                                  quietly : gen b = (`n'^2)/(((`n'*(`l2vc'))+(`l1vc'))^2)
     99.                                  quietly : gen p = 1 if `n' > 0 & `n' != .
    100.                                  collapse (sum) a b p, by(`l3var')
    101.                                  quietly: means p
    102.                                  scalar `p' = r(mean_h)
    103.                                  gen d = b/(1+(a*(`l3vc')))
    104.                                  gen e = (a^2)/(1+(a*(`l3vc')))
    105.                                  collapse (sum) d e
    106.                                  gen c = -1*((d*`l2vcv')/e)
    107.
 25 .                                  scalar `c23' = c
    108.
 26 .                                  display as text "Harmonic Mean of Level 2 Units per Level 3 Unit" _c
    109.                                  return scalar p = `p'
    110.                                  return scalar c23 = `c23'
    111.
 27 .                                  *variance of icc2
 28 .                                  scalar `v2' = ((((1-`icc2')^2)*`l2vcv')/`tv4')+(((`icc2'^2)*`l3vcv')
    112.                                  scalar `se2' = `v2'^.5
    113.
 29 .                                  *variance of icc3
 30 .
 31 .                                  scalar `v3' = (((`icc3'^2)*`l2vcv')/`tv4') + ((((1-`icc3')^2)*`l3vcv
    114.                                  scalar `se3' = `v3'^.5
    115.
 32 .                                  *covaraince?
 33 .
 34 .                                  scalar `cv23' = .
    116.
 35 .                                  matrix define `b' = (`icc3', `icc2')
    117.                                  matrix define `V' = (`v3', `cv23' \ `cv23', `v2')
    118.                                  matrix colnames `b' = `l3var' `l2var'
    119.                                  matrix rownames `b' = `y'
    120.                                  matrix colnames `V' = `l3var' `l2var'
    121.                                  matrix rownames `V' = `l3var' `l2var'
    122.                          }
    123.                          restore
    124.                  }
    125.                  else if `levels' == 3 {
    126.                          if "`unbalance'" == "unbalance" {
    127.                                  display as error "there is no balance option for four level model
    128.                                  exit 9
    129.                          }
    130.                          preserve
    131.                          gen `n' = e(sample)
    132.                          local lvars = e(ivars)
    133.                          local k = 4
    134.                          foreach v in `lvars' {
    135.                                  local l`k'var "`v'"
    136.                                  local --k
```

```
    137.                            }
    138.                            local y = e(depvar)
    139.                            quietly : _diparm lns1_1_1, function((exp(@))^2) derivative(2*exp(@)^2)
    140.                            scalar `l4vc' = r(est)
    141.                            scalar `l4vcv' = (r(se))^2
    142.                            quietly : _diparm lns2_1_1, function((exp(@))^2) derivative(2*exp(@)^2)
    143.                            scalar `l3vc' = r(est)
    144.                            scalar `l3vcv' = (r(se))^2
    145.                            quietly : _diparm lns3_1_1, function((exp(@))^2) derivative(2*exp(@)^2)
    146.                            scalar `l2vc' = r(est)
    147.                            scalar `l2vcv' = (r(se))^2
    148.                            quietly : _diparm lnsig_e, function((exp(@))^2) derivative(2*exp(@)^2)
    149.                            scalar `l1vc' = r(est)
    150.                            scalar `tv' = `l1vc' + `l2vc' + `l3vc' + `l4vc'
    151.                            scalar `tv4' = ((`tv')^.5)^4
    152.                            scalar `icc2' = `l2vc' / `tv'
    153.                            scalar `icc3' = `l3vc' / `tv'
    154.                            scalar `icc4' = `l4vc' / `tv'
    155.                            scalar `icc4bar' = 1-`icc4'
    156.                            scalar `icc3bar' = 1-`icc3'
    157.                            scalar `icc2bar' = 1-`icc2'
    158.
 36 .
 37 .                       collapse (max) `n' if e(sample), by(`l2var' `l3var' `l4var')
    159.                            quietly drop if `n' == 0
    160.                            collapse (sum) `n', by(`l3var' `l4var')
    161.                            quietly: means `n'
    162.                            scalar `p' = r(mean_h)
    163.                            quietly replace `n' = 1
    164.                            collapse (sum) `n', by( `l4var')
    165.                            quietly: means `n'
    166.                            scalar `q' = r(mean_h)
    167.
 38 .                       display as text "Harmonic Mean of Level 2 Units per Level 3 Unit" _col(50) "
    168.                            return scalar p = `p'
    169.                            display as text "Harmonic Mean of Level 3 Units per Level 4 Unit" _col(5
    170.                            return scalar q = `q'
    171.
 39 .                       scalar `qp' = `q'*`p'
    172.                            scalar `qp2' = `q'*(`p'^2)
    173.                            scalar `qp2tv4' = `q'*(`p'^2)*`tv4'
    174.                            scalar `qtv4' = `q'*`tv4'
    175.
 40 .                       *variance of icc2
 41 .
 42 .                       scalar `v2' = ((((`qp2'*(`icc2bar'^2))+(2*`qp'*`icc2'*`icc2bar')+(2*(`icc2'^
    >                                                   + (((`q'-2)*(`icc2'^2)*`l3vcv')/`qtv4') ///
    >                                                   + (((`icc2'^2)*`l4vcv')/`tv4')
    176.
 43 .                       scalar `se2' = `v2'^.5
    177.
 44 .                       *variance of icc3
 45 .
 46 .                       scalar `v3' = ((((`qp2'*(`icc3'^2))+(2*(`qp'-1)*`icc3'*`icc3bar'))*`l2vcv')/
    >                                                   + ((((`q'*(`icc3bar'^2))+(2*`icc3'*`icc3bar'))*`l3vc
    >                                                   + (((`icc3'^2)*`l4vcv')/`tv4')
    178.
 47 .                       scalar `se3' = `v3'^.5
    179.
 48 .                       *variance of icc4
 49 .
```

```
50 .                         scalar `v4' = ((((`qp'*(`p'-2)*`icc4'^2)-(2*`icc4'*`icc4bar'))*`l2vcv')/`qp2
   >                                                + ((((`q'*(`icc4'^2)) + (2*`icc4'*`icc4bar'))*`l3vcv
   >                                                + (((`icc4bar'^2)*`l4vcv')/`tv4')
   180.
51 .                         scalar `se4' = `v4'^.5
   181.
52 .
53 .                         *covariance between icc2 and icc3
54 .
55 .                         scalar `cv23' = ((((-`qp2'*`icc2bar'*`icc3')+(`qp'*((`icc2'*`icc3')-(`icc2ba
   > `l2vcv')/`qp2tv4') ///
   >                                                    - ((((`q'*`icc2'*`icc3bar')-(`icc2'*`icc3bar')+(`i
   >                                                    + ((`icc2'*`icc3'*`l4vcv')/`tv4')
   182.
56 .                         *covariance between icc2 and icc4
57 .
58 .                         scalar `cv24' = ((((-1*(`q'*(`p'^2)*`icc2bar'*`icc4'))+(`q'*`p'*((`icc2bar'*
   > cc4bar'))*`l2vcv')/(`q'*(`p'^2)*`tv4')) + ((((`q'*`icc2'*`icc4')-(`icc2'*`icc4')+(`icc2'*`icc4bar'))
   > /`tv4')
   183.
59 .                         *covariance between icc3 and icc4
60 .
61 .                         scalar `cv34' = ((((`qp2'*`icc3'*`icc4')+(`qp'*((`icc3bar'*`icc4')-(`icc3'*`
   > cv')/`qp2tv4') ///
   >                                                    - ((((`q'*`icc3bar'*`icc4')+(`icc3'*`icc4')+(`icc3
   >                                                    - ((`icc3'*`icc4bar'*`l4vcv')/`tv4')
   184.
62 .                         matrix define `b' = (`icc4', `icc3', `icc2')
   185.                         matrix define `V' = (`v4', `cv34' , `cv24' \ `cv34', `v3' , `cv23' \ `cv2
   186.                         matrix colnames `b' = `l4var' `l3var' `l2var'
   187.                         matrix rownames `b' = `y'
   188.                         matrix colnames `V' = `l4var' `l3var' `l2var'
   189.                         matrix rownames `V' = `l4var' `l3var' `l2var'
   190.
63 .                         restore
   191.
64 .                 }
   192.
65 .                 *post results
66 .
67 .                 return local model = e(cmdline)
   193.                 return scalar tv = `tv'
   194.                 return scalar l1vc = `l1vc'
   195.                 return matrix b = `b'
   196.                 return matrix V = `V'
   197.
68 .
69 .                 *display results & calculate ci
70 .
71 .                 scalar `z' = invnormal(1-(`alpha'/2))
   198.
72 .                 local lv = `levels'+1
   199.
73 .                 display _newline as text "{hline 13}" "{c TT}" "{hline 51}"
   200.                 display as text _col(14) "{c |}" _col(19) "ICC" _col(28) "Std. Err." ///
   >                 _col(43) %12.0f "[" (1-`alpha')*100 "% Conf. Interval]"
   201.                 display as text "{hline 13}" "{c +}" "{hline 51}"
   202.
74 .                 forvalues level = `lv'(-1)2 {
   203.                         return scalar l`level'vc = `l`level'vc'
   204.                         return scalar l`level'vc_v = `l`level'vcv'
   205.                         scalar `lower' = `icc`level'' - (`z'*`se`level'')
   206.                         scalar `higher' = `icc`level'' + (`z'*`se`level'')
   207.                         if `lower' < 0 {
   208.                                 scalar `lower' = 0
   209.                         }
   210.                         if `higher' > 1 {
   211.                                 scalar `higher' = 1
   212.                         }
   213.                         display as text as text %12s abbrev("`l`level'var'",12) _col(14) "{c |}"
   >                         _col(15) as result %9.5f `icc`level'' _col(27) as result %9.5f `se`level'' _
   >                         as result %9.5f `lower' _col(54) as result %9.5f `higher'
   214.                 }
```

```
   215.                 display as text "{hline 13}" "{c BT}" "{hline 51}"
   216.
75 .
76 .         }
   217.         else {
   218.                 display as error "can't find last xtmixed or mixed estimations"
   219.                 exit 301
   220.         }
   221. end

77 .
   end of do-file

78 .
   end of do-file

79 . do "C:\Users\zkuz0001\AppData\Local\Temp\STD00000000.tmp"

80 . cd "C:\! PRIVATE TO KEEP\! UNI TEMP\1290 # M_123_2016_04_08 # 40% EtbS\ADJUSTED PAPER\DATA NO OUTLIE
   C:\! PRIVATE TO KEEP\! UNI TEMP\1290 # M_123_2016_04_08 # 40% EtbS\ADJUSTED PAPER\DATA NO OUTLIE

81 .
   end of do-file

82 . do "C:\Users\zkuz0001\AppData\Local\Temp\STD00000000.tmp"

83 . use "Data_2016_05_21_NO OUTLIER_Mean_0.dta", clear

84 .
   end of do-file

85 . do "C:\Users\zkuz0001\AppData\Local\Temp\STD00000000.tmp"

86 . mixed MRD || Patient_ID: || Side: || Sample:, vce(robust) level(99)

   Performing EM optimization:

   Performing gradient-based optimization:

   Iteration 0:   log pseudolikelihood =   -23.276431
   Iteration 1:   log pseudolikelihood =   -23.117876
   Iteration 2:   log pseudolikelihood =   -23.117502
   Iteration 3:   log pseudolikelihood =   -23.117502

   Computing standard errors:

   Mixed-effects regression                         Number of obs     =         44
```

Group Variable	No. of Groups	Observations per Group Minimum	Average	Maximum
**Patient_ID**	**6**	**4**	**7.3**	**8**
**Side**	**12**	**2**	**3.7**	**4**
**Sample**	**24**	**1**	**1.8**	**2**

```
                                                 Wald chi2(    0)     =          .
   Log pseudolikelihood =  -23.117502              Prob > chi2       =          .

                              (Std. Err. adjusted for   6 clusters in Patient_ID)
```

MRD	Coef.	Robust Std. Err.	z	P>|z|	[99% Conf.	Interval]
_cons	**-5.246454**	**.0933276**	**-56.22**	**0.000**	**-5.48685**	**-5.006058**

Random-effects Parameters	Estimate	Robust Std. Err.	[99% Conf.	Interval]
**Patient_ID**: Identity				
var(_cons)	**2.18e-21**	.	.	.
**Side**: Identity				
var(_cons)	**.0917021**	**.0554169**	**.0193354**	**.4349172**
**Sample**: Identity				
var(_cons)	**.0388618**	**.0177326**	**.0119971**	**.1258841**
var(Residual)	**.0902209**	**.0192675**	**.0520482**	**.1563899**

87 . iccvar

Intraclass Correlation Estimates

Harmonic Mean of Level 2 Units per Level 3 Unit = **2.000**
Harmonic Mean of Level 3 Units per Level 4 Unit = **2.000**

	ICC	Std. Err.	[99% Conf.	Interval]
Patient_ID	**0.00000**	.	.	**1.00000**
Side	**0.41535**	.	.	**1.00000**
Sample	**0.17602**	.	.	**1.00000**

88 . use "Data_2016_05_21_NO OUTLIER_Mean_1.dta", clear

89 . mixed MRD || Patient_ID: || Side: || Sample:, vce(robust) level(99)

Performing EM optimization:

Performing gradient-based optimization:

Iteration 0: log pseudolikelihood = **-62.343323**
Iteration 1: log pseudolikelihood = **-62.241476**
Iteration 2: log pseudolikelihood = **-62.241458**
Iteration 3: log pseudolikelihood = **-62.241458**

Computing standard errors:

Mixed-effects regression Number of obs = **134**

Group Variable	No. of Groups	Observations per Group Minimum	Average	Maximum
**Patient_ID**	**17**	**7**	**7.9**	**8**
**Side**	**34**	**3**	**3.9**	**4**
**Sample**	**68**	**1**	**2.0**	**2**

Wald chi2(**0**) = .
Log pseudolikelihood = **-62.241458** Prob > chi2 = .

(Std. Err. adjusted for **17** clusters in Patient_ID)

MRD	Coef.	Robust Std. Err.	z	P>|z|	[99% Conf.	Interval]
_cons	**-3.852961**	**.1568333**	**-24.57**	**0.000**	**-4.256937**	**-3.448985**

Random-effects Parameters	Estimate	Robust Std. Err.	[99% Conf.	Interval]
**Patient_ID**: Identity				
var(_cons)	.3526466	.0802752	.196196	.6338542
**Side**: Identity				
var(_cons)	.0543047	.0339989	.010826	.2724013
**Sample**: Identity				
var(_cons)	.0215573	.0203995	.0018837	.2467061
var(Residual)	.0654505	.0232219	.0262427	.1632367

```
90 . iccvar
```

Intraclass Correlation Estimates

Harmonic Mean of Level 2 Units per Level 3 Unit = **2.000**
Harmonic Mean of Level 3 Units per Level 4 Unit = **2.000**

	ICC	Std. Err.	[99% Conf.	Interval]
Patient_ID	**0.71392**	**0.07387**	**0.52364**	**0.90419**
Side	**0.10994**	**0.06842**	**0.00000**	**0.28618**
Sample	**0.04364**	**0.04101**	**0.00000**	**0.14929**

```
91 . use "Data_2016_05_21_NO OUTLIER_Mean_2.dta", clear
92 . mixed MRD || Patient_ID: || Side: || Sample:, vce(robust) level(99)
```

Performing EM optimization:

Performing gradient-based optimization:

Iteration 0: log pseudolikelihood = **-14.077809**
Iteration 1: log pseudolikelihood = **-13.847935**
Iteration 2: log pseudolikelihood = **-13.840535**
Iteration 3: log pseudolikelihood = **-13.840445**
Iteration 4: log pseudolikelihood = **-13.840445**

Computing standard errors:

Mixed-effects regression Number of obs = **40**

Group Variable	No. of Groups	Observations per Group Minimum	Average	Maximum
**Patient_ID**	**5**	**8**	**8.0**	**8**
**Side**	**10**	**4**	**4.0**	**4**
**Sample**	**20**	**2**	**2.0**	**2**

Wald chi2(**0**) = **.**
Log pseudolikelihood = **-13.840445** Prob > chi2 = **.**

(Std. Err. adjusted for **5** clusters in Patient_ID)

MRD	Coef.	Robust Std. Err.	z	P>|z|	[99% Conf.	Interval]
_cons	**-2.116806**	**.1720275**	**-12.31**	**0.000**	**-2.559919**	**-1.673693**

Random-effects Parameters	Estimate	Robust Std. Err.	[99% Conf.	Interval]
**Patient_ID**: Identity				
var(_cons)	.1049092	.0762204	.0161458	.6816598
**Side**: Identity				
var(_cons)	3.17e-17	6.06e-16	1.26e-38	79855.94
**Sample**: Identity				
var(_cons)	.0169771	.0221417	.00059	.4884749
var(Residual)	.0737625	.0269776	.028754	.1892224

93 . iccvar

Intraclass Correlation Estimates

```
Harmonic Mean of Level 2 Units per Level 3 Unit  =          2.000
Harmonic Mean of Level 3 Units per Level 4 Unit  =          2.000
```

	ICC	Std. Err.	[99% Conf.	Interval]
Patient_ID	0.53621	0.17846	0.07652	0.99591
Side	0.00000	0.00000	0.00000	0.00000
Sample	0.08677	0.11342	0.00000	0.37891

94 . use "Data_2016_05_21_NO OUTLIER_Median_0.dta", clear

95 . mixed MRD || Patient_ID: || Side: || Sample:, vce(robust) level(99)

Performing EM optimization:

Performing gradient-based optimization:

```
Iteration 0:   log pseudolikelihood =   -26.674552
Iteration 1:   log pseudolikelihood =   -26.235434
Iteration 2:   log pseudolikelihood =   -26.217235
Iteration 3:   log pseudolikelihood =   -26.217146
Iteration 4:   log pseudolikelihood =   -26.217146
```

Computing standard errors:

Mixed-effects regression   Number of obs   =   43

Group Variable	No. of Groups	Observations per Group Minimum	Average	Maximum
**Patient_ID**	6	4	7.2	8
**Side**	12	2	3.6	4
**Sample**	24	1	1.8	2

```
                                            Wald chi2(    0)      =           .
Log pseudolikelihood =  -26.217146            Prob > chi2        =           .
```

(Std. Err. adjusted for 6 clusters in Patient_ID)

MRD	Coef.	Robust Std. Err.	z	P>|z|	[99% Conf.	Interval]
_cons	-5.218633	.1037167	-50.32	0.000	-5.485789	-4.951476

Random-effects Parameters	Estimate	Robust Std. Err.	[99% Conf.	Interval]
**Patient_ID**: Identity				
var(_cons)	**1.86e-19**	**.**	**.**	**.**
**Side**: Identity				
var(_cons)	**.0920649**	**.0549646**	**.01978**	**.4285105**
**Sample**: Identity				
var(_cons)	**6.91e-23**	**1.53e-20**	**4.2e-271**	**1.1e+226**
var(Residual)	**.1423424**	**.0378454**	**.0717641**	**.2823324**

96 . iccvar

Intraclass Correlation Estimates

Harmonic Mean of Level 2 Units per Level 3 Unit = **2.000**
Harmonic Mean of Level 3 Units per Level 4 Unit = **2.000**

	ICC	Std. Err.	[99% Conf.	Interval]
Patient_ID	**0.00000**	**.**	**.**	**1.00000**
Side	**0.39276**	**.**	**.**	**1.00000**
Sample	**0.00000**	**.**	**.**	**1.00000**

97 . use "Data_2016_05_21_NO OUTLIER_Median_1.dta", clear

98 . mixed MRD || Patient_ID: || Side: || Sample:, vce(robust) level(99)

Performing EM optimization:

Performing gradient-based optimization:

Iteration 0: log pseudolikelihood = **-48.42102**
Iteration 1: log pseudolikelihood = **-48.399884**
Iteration 2: log pseudolikelihood = **-48.399877**
Iteration 3: log pseudolikelihood = **-48.399877**

Computing standard errors:

Mixed-effects regression Number of obs = **127**

Group Variable	No. of Groups	Observations per Group Minimum	Average	Maximum
**Patient_ID**	**16**	**7**	**7.9**	**8**
**Side**	**32**	**3**	**4.0**	**4**
**Sample**	**64**	**1**	**2.0**	**2**

Wald chi2(**0**) = **.**
Log pseudolikelihood = **-48.399877** Prob > chi2 = **.**

(Std. Err. adjusted for **16** clusters in Patient_ID)

MRD	Coef.	Robust Std. Err.	z	P>|z|	[99% Conf.	Interval]
_cons	**-3.913159**	**.1625213**	**-24.08**	**0.000**	**-4.331786**	**-3.494532**

Random-effects Parameters	Estimate	Robust Std. Err.	[99% Conf.	Interval]
**Patient_ID**: Identity				
var(_cons)	**.3608389**	**.0912117**	**.1881655**	**.6919691**
**Side**: Identity				
var(_cons)	**.0433773**	**.0304153**	**.0071265**	**.2640288**
**Sample**: Identity				
var(_cons)	**.0300258**	**.0145008**	**.0086545**	**.1041715**
var(Residual)	**.0487096**	**.0114392**	**.0266011**	**.0891927**

99 . iccvar

Intraclass Correlation Estimates

Harmonic Mean of Level 2 Units per Level 3 Unit = **2.000**
Harmonic Mean of Level 3 Units per Level 4 Unit = **2.000**

	ICC	Std. Err.	[99% Conf.	Interval]
Patient_ID	**0.74715**	**0.07212**	**0.56139**	**0.93292**
Side	**0.08982**	**0.06293**	**0.00000**	**0.25192**
Sample	**0.06217**	**0.03137**	**0.00000**	**0.14298**

100 . use "Data_2016_05_21_NO OUTLIER_Median_2.dta", clear

101 . mixed MRD || Patient_ID: || Side: || Sample:, vce(robust) level(99)

Performing EM optimization:

Performing gradient-based optimization:

Iteration 0: log pseudolikelihood = **-22.891868**
Iteration 1: log pseudolikelihood = **-22.887461**
Iteration 2: log pseudolikelihood = **-22.88746**

Computing standard errors:

Mixed-effects regression Number of obs = **48**

Group Variable	No. of Groups	Observations per Group Minimum	Average	Maximum
**Patient_ID**	**6**	**8**	**8.0**	**8**
**Side**	**12**	**4**	**4.0**	**4**
**Sample**	**24**	**2**	**2.0**	**2**

Wald chi2(**0**) = **.**
Log pseudolikelihood = **-22.88746** Prob > chi2 = **.**

(Std. Err. adjusted for **6** clusters in Patient_ID)

MRD	Coef.	Robust Std. Err.	z	P>|z|	[99% Conf.	Interval]
_cons	**-2.270639**	**.208311**	**-10.90**	**0.000**	**-2.807212**	**-1.734065**

Random-effects Parameters	Estimate	Robust Std. Err.	[99% Conf.	Interval]
**Patient_ID**: Identity				
var(_cons)	**.1692455**	**.0541276**	**.0742588**	**.3857326**
**Side**: Identity				
var(_cons)	**.0577363**	**.0629971**	**.0034742**	**.9595075**
**Sample**: Identity				
var(_cons)	**.0424705**	**.0288436**	**.007385**	**.2442451**
var(Residual)	**.0658885**	**.0233921**	**.026403**	**.1644244**

102 . iccvar

Intraclass Correlation Estimates

Harmonic Mean of Level 2 Units per Level 3 Unit = **2.000**
Harmonic Mean of Level 3 Units per Level 4 Unit = **2.000**

	ICC	Std. Err.	[99% Conf.	Interval]
Patient_ID	**0.50470**	**0.15408**	**0.10781**	**0.90158**
Side	**0.17217**	**0.17606**	**0.00000**	**0.62568**
Sample	**0.12665**	**0.08312**	**0.00000**	**0.34075**

103 .
end of do-file

104 . do "C:\Users\zkuz0001\AppData\Local\Temp\STD00000000.tmp"

105 . cd "C:\! PRIVATE TO KEEP\! UNI TEMP\1290 # M_123_2016_04_08 # 40% EtbS\ADJUSTED PAPER\DATA ALL CASES
C:\! PRIVATE TO KEEP\! UNI TEMP\1290 # M_123_2016_04_08 # 40% EtbS\ADJUSTED PAPER\DATA ALL CASES

106 .
end of do-file

107 . do "C:\Users\zkuz0001\AppData\Local\Temp\STD00000000.tmp"

108 . use "Data_2016_05_21_ALL CASES_Mean_0.dta", clear

109 . mixed MRD || Patient_ID: || Side: || Sample:, vce(robust) level(99)

Performing EM optimization:

Performing gradient-based optimization:

Iteration 0: log pseudolikelihood = **-23.276431**
Iteration 1: log pseudolikelihood = **-23.117876**
Iteration 2: log pseudolikelihood = **-23.117502**
Iteration 3: log pseudolikelihood = **-23.117502**

Computing standard errors:

Mixed-effects regression Number of obs = **44**

Group Variable	No. of Groups	Observations per Group Minimum	Average	Maximum
**Patient_ID**	**6**	**4**	**7.3**	**8**
**Side**	**12**	**2**	**3.7**	**4**
**Sample**	**24**	**1**	**1.8**	**2**

Wald chi2(**0**) = .
Log pseudolikelihood = **-23.117502** Prob > chi2 = .

(Std. Err. adjusted for **6** clusters in Patient_ID)

MRD	Coef.	Robust Std. Err.	z	P>|z|	[99% Conf.	Interval]
_cons	**-5.246454**	**.0933276**	**-56.22**	**0.000**	**-5.48685**	**-5.006058**

Random-effects Parameters	Estimate	Robust Std. Err.	[99% Conf.	Interval]
**Patient_ID**: Identity				
var(_cons)	**2.18e-21**	**.**	**.**	**.**
**Side**: Identity				
var(_cons)	**.0917021**	**.0554169**	**.0193354**	**.4349172**
**Sample**: Identity				
var(_cons)	**.0388618**	**.0177326**	**.0119971**	**.1258841**
var(Residual)	**.0902209**	**.0192675**	**.0520482**	**.1563899**

110 . iccvar

Intraclass Correlation Estimates

Harmonic Mean of Level 2 Units per Level 3 Unit = **2.000**
Harmonic Mean of Level 3 Units per Level 4 Unit = **2.000**

	ICC	Std. Err.	[99% Conf.	Interval]
Patient_ID	**0.00000**	**.**	**.**	**1.00000**
Side	**0.41535**	**.**	**.**	**1.00000**
Sample	**0.17602**	**.**	**.**	**1.00000**

111 . use "Data_2016_05_21_ALL CASES_Mean_1.dta", clear

112 . mixed MRD || Patient_ID: || Side: || Sample:, vce(robust) level(99)

Performing EM optimization:

Performing gradient-based optimization:

```
Iteration 0:   log pseudolikelihood =   -62.343323
Iteration 1:   log pseudolikelihood =   -62.241476
Iteration 2:   log pseudolikelihood =   -62.241458
Iteration 3:   log pseudolikelihood =   -62.241458
```

Computing standard errors:

Mixed-effects regression   Number of obs   =   **134**

Group Variable	No. of Groups	Observations per Group Minimum	Average	Maximum
**Patient_ID**	**17**	**7**	**7.9**	**8**
**Side**	**34**	**3**	**3.9**	**4**
**Sample**	**68**	**1**	**2.0**	**2**

Wald chi2(**0**) = **.**
Log pseudolikelihood = **-62.241458**   Prob > chi2 = **.**

(Std. Err. adjusted for **17** clusters in Patient_ID)

MRD	Coef.	Robust Std. Err.	z	P>|z|	[99% Conf.	Interval]
_cons	**-3.852961**	**.1568333**	**-24.57**	**0.000**	**-4.256937**	**-3.448985**

Random-effects Parameters	Estimate	Robust Std. Err.	[99% Conf.	Interval]
**Patient_ID**: Identity				
var(_cons)	**.3526466**	**.0802752**	**.196196**	**.6338542**
**Side**: Identity				
var(_cons)	**.0543047**	**.0339989**	**.010826**	**.2724013**
**Sample**: Identity				
var(_cons)	**.0215573**	**.0203995**	**.0018837**	**.2467061**
var(Residual)	**.0654505**	**.0232219**	**.0262427**	**.1632367**

113 . iccvar

Intraclass Correlation Estimates

Harmonic Mean of Level 2 Units per Level 3 Unit = **2.000**
Harmonic Mean of Level 3 Units per Level 4 Unit = **2.000**

	ICC	Std. Err.	[99% Conf.	Interval]
Patient_ID	**0.71392**	**0.07387**	**0.52364**	**0.90419**
Side	**0.10994**	**0.06842**	**0.00000**	**0.28618**
Sample	**0.04364**	**0.04101**	**0.00000**	**0.14929**

114 . use "Data_2016_05_21_ALL CASES_Mean_2.dta", clear

115 . mixed MRD || Patient_ID: || Side: || Sample:, vce(robust) level(99)

Performing EM optimization:

Performing gradient-based optimization:

Iteration 0: log pseudolikelihood = **-33.039049**
Iteration 1: log pseudolikelihood = **-32.768493**
Iteration 2: log pseudolikelihood = **-32.76245**
Iteration 3: log pseudolikelihood = **-32.762446**
Iteration 4: log pseudolikelihood = **-32.762446**

Computing standard errors:

Mixed-effects regression   Number of obs = **48**

Group Variable	No. of Groups	Observations per Group Minimum	Average	Maximum
**Patient_ID**	**6**	**8**	**8.0**	**8**
**Side**	**12**	**4**	**4.0**	**4**
**Sample**	**24**	**2**	**2.0**	**2**

Wald chi2(**0**) = **.**
Log pseudolikelihood = **-32.762446**   Prob > chi2 = **.**

(Std. Err. adjusted for **6** clusters in Patient_ID)

MRD	Coef.	Robust Std. Err.	z	P>|z|	[99% Conf.	Interval]
_cons	**-2.106453**	**.1408409**	**-14.96**	**0.000**	**-2.469235**	**-1.743671**

Random-effects Parameters	Estimate	Robust Std. Err.	[99% Conf.	Interval]
**Patient_ID**: Identity				
var(_cons)	**1.90e-24**	**6.32e-22**	**0**	**.**
**Side**: Identity				
var(_cons)	**.5690522**	**.9438939**	**.007936**	**40.80388**
**Sample**: Identity				
var(_cons)	**.0100111**	**.0987663**	**9.21e-14**	**1.09e+09**
var(Residual)	**.0986263**	**.1493614**	**.0019946**	**4.876677**

116 . iccvar

Intraclass Correlation Estimates

Harmonic Mean of Level 2 Units per Level 3 Unit = **2.000**
Harmonic Mean of Level 3 Units per Level 4 Unit = **2.000**

	ICC	Std. Err.	[99% Conf.	Interval]
Patient_ID	**0.00000**	**0.00000**	**0.00000**	**0.00000**
Side	**0.83969**	**0.57280**	**0.00000**	**1.00000**
Sample	**0.01477**	**0.14466**	**0.00000**	**0.38740**

117 . use "Data_2016_05_21_ALL CASES_Median_0.dta", clear

118 . mixed MRD || Patient_ID: || Side: || Sample:, vce(robust) level(99)

Performing EM optimization:

Performing gradient-based optimization:

Iteration 0: log pseudolikelihood = **-26.674552**
Iteration 1: log pseudolikelihood = **-26.235434**
Iteration 2: log pseudolikelihood = **-26.217235**
Iteration 3: log pseudolikelihood = **-26.217146**
Iteration 4: log pseudolikelihood = **-26.217146**

Computing standard errors:

Mixed-effects regression   Number of obs = **43**

Group Variable	No. of Groups	Observations per Group Minimum	Average	Maximum
**Patient_ID**	**6**	**4**	**7.2**	**8**
**Side**	**12**	**2**	**3.6**	**4**
**Sample**	**24**	**1**	**1.8**	**2**

Wald chi2(**0**) = **.**
Log pseudolikelihood = **-26.217146**   Prob > chi2 = **.**

(Std. Err. adjusted for **6** clusters in Patient_ID)

MRD	Coef.	Robust Std. Err.	z	P>|z|	[99% Conf.	Interval]
_cons	**-5.218633**	**.1037167**	**-50.32**	**0.000**	**-5.485789**	**-4.951476**

Random-effects Parameters	Estimate	Robust Std. Err.	[99% Conf.	Interval]
**Patient_ID**: Identity				
var(_cons)	**1.86e-19**	**.**	**.**	**.**
**Side**: Identity				
var(_cons)	**.0920649**	**.0549646**	**.01978**	**.4285105**
**Sample**: Identity				
var(_cons)	**6.91e-23**	**1.53e-20**	**4.2e-271**	**1.1e+226**
var(Residual)	**.1423424**	**.0378454**	**.0717641**	**.2823324**

119 . iccvar

Intraclass Correlation Estimates

Harmonic Mean of Level 2 Units per Level 3 Unit = **2.000**
Harmonic Mean of Level 3 Units per Level 4 Unit = **2.000**

	ICC	Std. Err.	[99% Conf.	Interval]
Patient_ID	**0.00000**	**.**	**.**	**1.00000**
Side	**0.39276**	**.**	**.**	**1.00000**
Sample	**0.00000**	**.**	**.**	**1.00000**

120 . use "Data_2016_05_21_ALL CASES_Median_1.dta", clear

121 . mixed MRD || Patient_ID: || Side: || Sample:, vce(robust) level(99)

Performing EM optimization:

Performing gradient-based optimization:

Iteration 0: log pseudolikelihood = **-48.42102**
Iteration 1: log pseudolikelihood = **-48.399884**
Iteration 2: log pseudolikelihood = **-48.399877**
Iteration 3: log pseudolikelihood = **-48.399877**

Computing standard errors:

Mixed-effects regression   Number of obs = **127**

Group Variable	No. of Groups	Observations per Group Minimum	Average	Maximum
**Patient_ID**	**16**	**7**	**7.9**	**8**
**Side**	**32**	**3**	**4.0**	**4**
**Sample**	**64**	**1**	**2.0**	**2**

Wald chi2(**0**) = **.**
Log pseudolikelihood = **-48.399877**   Prob > chi2 = **.**

(Std. Err. adjusted for **16** clusters in Patient_ID)

MRD	Coef.	Robust Std. Err.	z	P>|z|	[99% Conf. Interval]	
_cons	**-3.913159**	**.1625213**	**-24.08**	**0.000**	**-4.331786**	**-3.494532**

Random-effects Parameters	Estimate	Robust Std. Err.	[99% Conf. Interval]	
**Patient_ID**: Identity				
var(_cons)	**.3608389**	**.0912117**	**.1881655**	**.6919691**
**Side**: Identity				
var(_cons)	**.0433773**	**.0304153**	**.0071265**	**.2640288**
**Sample**: Identity				
var(_cons)	**.0300258**	**.0145008**	**.0086545**	**.1041715**
var(Residual)	**.0487096**	**.0114392**	**.0266011**	**.0891927**

122 . iccvar

Intraclass Correlation Estimates

```
Harmonic Mean of Level 2 Units per Level 3 Unit  =          2.000
Harmonic Mean of Level 3 Units per Level 4 Unit  =          2.000
```

	ICC	Std. Err.	[99% Conf. Interval]	
Patient_ID	**0.74715**	**0.07212**	**0.56139**	**0.93292**
Side	**0.08982**	**0.06293**	**0.00000**	**0.25192**
Sample	**0.06217**	**0.03137**	**0.00000**	**0.14298**

123 . use "Data_2016_05_21_ALL CASES_Median_2.dta", clear

124 . mixed MRD || Patient_ID: || Side: || Sample:, vce(robust) level(99)

Performing EM optimization:

Performing gradient-based optimization:

```
Iteration 0:   log pseudolikelihood =   -39.070963
Iteration 1:   log pseudolikelihood =   -38.907314
Iteration 2:   log pseudolikelihood =   -38.906968
Iteration 3:   log pseudolikelihood =   -38.906968
```

Computing standard errors:

Mixed-effects regression   Number of obs   =   **56**

Group Variable	No. of Groups	Observations per Group Minimum	Average	Maximum
**Patient_ID**	**7**	**8**	**8.0**	**8**
**Side**	**14**	**4**	**4.0**	**4**
**Sample**	**28**	**2**	**2.0**	**2**

```
                                            Wald chi2(    0)      =          .
Log pseudolikelihood =  -38.906968            Prob > chi2         =          .
```

(Std. Err. adjusted for **7** clusters in Patient_ID)

MRD	Coef.	Robust Std. Err.	z	P>|z|	[99% Conf.	Interval]
_cons	**-2.239788**	**.1787374**	**-12.53**	**0.000**	**-2.700186**	**-1.779391**

Random-effects Parameters	Estimate	Robust Std. Err.	[99% Conf.	Interval]
**Patient_ID**: Identity				
var(_cons)	**4.83e-21**	**1.33e-18**	**0**	**5.3e+288**
**Side**: Identity				
var(_cons)	**.6147788**	**.6739165**	**.0365125**	**10.35133**
**Sample**: Identity				
var(_cons)	**.0307096**	**.0377186**	**.001298**	**.7265405**
var(Residual)	**.0883253**	**.0733555**	**.0103995**	**.7501635**

125 . iccvar

Intraclass Correlation Estimates

Harmonic Mean of Level 2 Units per Level 3 Unit = **2.000**
Harmonic Mean of Level 3 Units per Level 4 Unit = **2.000**

	ICC	Std. Err.	[99% Conf.	Interval]
Patient_ID	**0.00000**	**0.00000**	**0.00000**	**0.00000**
Side	**0.83779**	**0.37274**	**0.00000**	**1.00000**
Sample	**0.04185**	**0.05033**	**0.00000**	**0.17148**

126 . use "Data_2016_05_21_ALL CASES.dta", clear

127 . mixed MRD || Patient_ID: || Side: || Sample:, vce(robust) level(99)

Performing EM optimization:

Performing gradient-based optimization:

Iteration 0: log pseudolikelihood = **-154.12226**
Iteration 1: log pseudolikelihood = **-153.8733**
Iteration 2: log pseudolikelihood = **-153.87326**
Iteration 3: log pseudolikelihood = **-153.87326**

Computing standard errors:

Mixed-effects regression   Number of obs = **226**

Group Variable	No. of Groups	Observations per Group Minimum	Average	Maximum
**Patient_ID**	**29**	**4**	**7.8**	**8**
**Side**	**58**	**2**	**3.9**	**4**
**Sample**	**116**	**1**	**1.9**	**2**

Wald chi2(**0**) = **.**
Log pseudolikelihood = **-153.87326**   Prob > chi2 = **.**

(Std. Err. adjusted for **29** clusters in Patient_ID)

MRD	Coef.	Robust Std. Err.	z	P>|z|	[99% Conf.	Interval]
_cons	**-3.780348**	**.2145761**	**-17.62**	**0.000**	**-4.333059**	**-3.227637**

Random-effects Parameters	Estimate	Robust Std. Err.	[99% Conf.	Interval]
**Patient_ID**: Identity				
var(_cons)	**1.142745**	**.2292444**	**.6816079**	**1.91586**
**Side**: Identity				
var(_cons)	**.2613908**	**.2015051**	**.035885**	**1.904002**
**Sample**: Identity				
var(_cons)	**.0225128**	**.0127965**	**.0052067**	**.0973403**
var(Residual)	**.0774167**	**.0157381**	**.0458584**	**.1306924**

128 . iccvar

Intraclass Correlation Estimates

Harmonic Mean of Level 2 Units per Level 3 Unit = **2.000**
Harmonic Mean of Level 3 Units per Level 4 Unit = **2.000**

	ICC	Std. Err.	[99% Conf.	Interval]
Patient_ID	**0.75977**	**0.12237**	**0.44457**	**1.00000**
Side	**0.17379**	**0.12466**	**0.00000**	**0.49490**
Sample	**0.01497**	**0.00875**	**0.00000**	**0.03750**

129 .
end of do-file

130 . do "C:\Users\zkuz0001\AppData\Local\Temp\STD00000000.tmp"

131 . cd "C:\! PRIVATE TO KEEP\! UNI TEMP\1290 # M_123_2016_04_08 # 40% EtbS\ADJUSTED PAPER\DATA NO OUTLIE
C:\! PRIVATE TO KEEP\! UNI TEMP\1290 # M_123_2016_04_08 # 40% EtbS\ADJUSTED PAPER\DATA NO OUTLIE

132 .
end of do-file

133 . do "C:\Users\zkuz0001\AppData\Local\Temp\STD00000000.tmp"

134 . use "Data_2016_05_21_NO OUTLIER.dta", clear

135 . mixed MRD || Patient_ID: || Side: || Sample:, vce(robust) level(99)

Performing EM optimization:

Performing gradient-based optimization:

Iteration 0: log pseudolikelihood = **-127.78599**
Iteration 1: log pseudolikelihood = **-127.62458**
Iteration 2: log pseudolikelihood = **-127.62457**
Iteration 3: log pseudolikelihood = **-127.62457**

Computing standard errors:

Mixed-effects regression   Number of obs = **218**

Group Variable	No. of Groups	Observations per Group Minimum	Average	Maximum
**Patient_ID**	**28**	**4**	**7.8**	**8**
**Side**	**56**	**2**	**3.9**	**4**
**Sample**	**112**	**1**	**1.9**	**2**

Wald chi2(**0**) = .
Log pseudolikelihood = **-127.62457** Prob > chi2 = .

(Std. Err. adjusted for **28** clusters in Patient_ID)

MRD	Coef.	Robust Std. Err.	z	P>|z|	[99% Conf.	Interval]
_cons	**-3.841953**	**.2130011**	**-18.04**	**0.000**	**-4.390607**	**-3.293298**

Random-effects Parameters	Estimate	Robust Std. Err.	[99% Conf.	Interval]
**Patient_ID**: Identity				
var(_cons)	**1.178316**	**.2395179**	**.6980212**	**1.989093**
**Side**: Identity				
var(_cons)	**.0620443**	**.0308711**	**.0172223**	**.223518**
**Sample**: Identity				
var(_cons)	**.0248367**	**.0131076**	**.0063785**	**.0967099**
var(Residual)	**.0719257**	**.0153071**	**.041573**	**.1244393**

136 . iccvar

Intraclass Correlation Estimates

Harmonic Mean of Level 2 Units per Level 3 Unit = **2.000**
Harmonic Mean of Level 3 Units per Level 4 Unit = **2.000**

	ICC	Std. Err.	[99% Conf.	Interval]
Patient_ID	**0.88123**	**0.03033**	**0.80311**	**0.95935**
Side	**0.04640**	**0.02410**	**0.00000**	**0.10848**
Sample	**0.01857**	**0.01027**	**0.00000**	**0.04502**

137 .
end of do-file

138 .
